# Chemogenetic activation of PVN CRH neurons disrupts the estrous cycle and LH dynamics in female mice

**DOI:** 10.3389/fendo.2023.1322662

**Published:** 2024-01-09

**Authors:** Junru Yu, Xiao-Feng Li, Krasimira Tsaneva-Atanasova, Eder Zavala, Kevin T. O’Byrne

**Affiliations:** ^1^ Department of Women and Children’s Health, School of Life Course and Population Sciences, Faculty of Life Science and Medicine, King’s College London, London, United Kingdom; ^2^ Department of Mathematics and Statistics, Faculty of Environment, Science and Economy, University of Exeter, Exeter, United Kingdom; ^3^ Living Systems Institute, University of Exeter, Exeter, United Kingdom; ^4^ Centre for Systems Modelling and Quantitative Biomedicine, University of Birmingham, Edgbaston, United Kingdom

**Keywords:** PVN, CRH, estrous cycle, LH, CORT, mathematical model

## Abstract

**Introduction:**

The impact of stress on reproductive function is significant. Hypothalamic paraventricular nucleus (PVN) corticotrophin-releasing hormone (CRH) plays a major role in regulating the stress response. Understanding how the hypothalamic-pituitary-adrenal (HPA) axis and the hypothalamic-pituitary-gonadal (HPG) axis interact is crucial for comprehending how stress can lead to reproductive dysfunction. However, whether stress influences reproductive function via modulating PVN CRH or HPA sequelae is not fully elucidated.

**Methods:**

In this study, we investigated the impact of chemogenetic activation of PVN CRH neurons on reproductive function. We chronically and selectively stimulated PVN CRH neurons in female CRH-Cre mice using excitatory designer receptor exclusively activated by designer drugs (DREADDs) viral constructs, which were bilaterally injected into the PVN. The agonist compound-21 (C21) was delivered through the drinking water. We determined the effects of DREADDs activation of PVN CRH neurons on the estrous cycles, LH pulse frequency in diestrus and metestrus and LH surge in proestrus mice. The effect of long-term C21 administration on basal corticosterone secretion and the response to acute restraint stress during metestrus was also examined. Additionally, computer simulations of a mathematical model were used to determine the effects of DREADDs activation of PVN CRH neurons, simulating chronic stress, on the physiological parameters examined experimentally.

**Results:**

As a result, and consistent with our mathematical model predictions, the length of the estrous cycle was extended, with an increase in the time spent in estrus and metestrus, and a decrease in proestrus and diestrus. Additionally, the frequency of LH pulses during metestrus was decreased, but unaffected during diestrus. The occurrence of the preovulatory LH surge during proestrus was disrupted. The basal level of corticosterone during metestrus was not affected, but the response to acute restraint stress was diminished after long-term C21 application.

**Discussion:**

These data suggest that PVN CRH neurons play a functional role in disrupting ovarian cyclicity and the preovulatory LH surge, and that the activity of the GnRH pulse generator remains relatively robust during diestrus but not during metestrus under chronic stress exposure in accordance with our mathematical model predictions.

## Introduction

In modern society, the occurrence of reproductive dysfunction is ever increasing, and stress is thought to be a common cause of infertility. The response to social and environmental stressors generally promotes survival while halting reproduction. A range of stress models depict a suppression in pulsatile LH secretion among various species (1). Stress can also impede the preovulatory GnRH/LH surge via delaying the rise in estradiol (E_2_) (2) or interfering with the ability of E_2_ to induce the GnRH/LH surge (3). In mice, chronic stress lengthens the estrous cycle and reduces the number of preovulatory follicles and corpora lutea (4). The impact of stress on the hypothalamic GnRH pulse generator and consequently on reproductive function is well described, but the underlying neural mechanism remains unclear (5).

In mammals, the hypothalamic-pituitary-adrenal (HPA) axis is the predominant neuroendocrine system activated in response to stressors (6, 7), with corticotropin-releasing hormone (CRH) being the primary initiating hypothalamic signal (6–9). The hypothalamic paraventricular nucleus (PVN) contains the most prominent CRH neuron population. Exposure to acute or chronic stressors increased circulating levels of ACTH and corticosterone (CORT), and inhibited plasma LH levels in rats. However, bilateral PVN lesions failed to block the stress-induced suppression of LH, while attenuating the adrenocorticotropic hormone (ACTH) and CORT response in rats (10). Nevertheless, more recent studies have shown that selective chemogenetic (11) or optogenetic (12) activation of PVN CRH neurons decreases LH pulse frequency in mice. Whether stress influences reproductive ovarian cycles and ovulation via modulating PVN CRH or via output from the HPA axis is not fully elucidated.

The reproductive cycle, and steroid hormones in particular, can influence stress hormone dynamics. Ovariectomized (OVX) rats showed an attenuated CORT circadian rhythm, which is reversed by E_2_ replacement (13). How the HPA and HPG axes coordinate their hormone rhythms is poorly understood. Since neuroendocrine axes are physiological regulatory systems involving multiple levels of organization and timescales combining sensitivity with robustness to perturbations, mathematical modeling has become a critical analytical tool to understand this complexity (14). We have previously developed a mathematical model proposing that the HPA and HPG axes interact as coupled oscillators (15). Several interesting predictions about the interplay between HPA and HPG axes were made, including that the rodent estrous cycle would be lengthened under prolonged CORT excess (a hallmark of chronic stress), and that disruption of the GnRH pulse generator frequency would be more susceptible to stressors in the estrus and metestrus phases but remain relatively robust during diestrus and early proestrus (15). While the mathematical model introduced the concept of “robustness” of the pulse generator as its ability to maintain or rapidly restore a steady frequency following stressors, little is known about how this property would be affected under chronic stress scenarios. Physiologically, this robustness might translate into a protective mechanism in later stages of follicular maturation and ovulation. These mathematical predictions are experimentally interrogated in the present study.

To do this, we used chemogenetic designer receptor exclusively activated by designer drugs (DREADDs) to selectively stimulate PVN CRH neurons, simulating chronic stress, to determine the effect on the estrous cycle, GnRH pulse generator frequency in diestrus and metestrus and LH surge in proestrus mice.

## Materials and methods

### Mice

CRH-cre-tdTomato transgenic mice were generated by breeding CRH-cre mice (Strain #: 012704, B6(Cg)-Crh^tm1(cre)Zjh^/J, Jackson Laboratory, Bar Harbour, ME, USA) with td-Tomato mice (Strain #:007909, B6.Cg-Gt(ROSA)26Sor^tm9(CAG-tdTomato)Hze^/J, Jackson Laboratory, Bar Harbor, ME, USA), to express the reporter allele encoding tdTomato upon Cre-mediated recombination. CRH-cre-tdTomato mice were genotyped using PCR to determine heterozygosity for CRH-cre (primers 5’-3’: Common, 10574, CTTACACATTTC GTCCTAGCC; Wild-type forward, 10575, ACGACCAGGCTGCGGCTAAC; Mutant forward, 105756, CAATGTATCTTATCATGTCTGGATCC) and tdTomato (primers 5’ - 3’: Wild type Forward oIMR9020 - AAGGGAGCTGCAGTGGAGTA; Wild type Reverse oIMR9021 - CCGAAAATCTGTGGGAAGTC; Mutant Reverse WPRE oIMR9103 - GGCATTAAAGCAGCGTATCC; Mutant Forward tdTomato oIMR9105 - CTGTTCCTGTACGGCATGG). Female mice weighing between 20-25g and aged 6-8 weeks were used. All animals were housed individually in ventilated cages at 25 ± 1°C in a 12:12-hour light-dark cycle, lights on at 07:00 h. The cages were equipped with wood-chip bedding and nesting material, food (standard maintenance diet; Special Dietary Services, Wittam, UK) and water ad libitum. All procedures were carried out following the United Kingdom Home Office Regulations and approved by the Animal Welfare and Ethical Review Body Committee at King’s College London.

### Stereotaxic injection of the DREADD viral construct

Animals were anesthetized using Ketamine (Vetalar, 100 mg/kg, i.p. injection; Pfizer, Sandwich, UK) and xylazine (Rompun, 10 mg/kg, i.p. injection; Bayer, Leverkusen, Germany). A robot stereotaxic system (Neurostar, Tubingen, Germany) was used to performed surgical procedures. The detailed stereotaxic coordinates used for bilateral viral injection into the PVN were obtained from the mouse brain atlas of Paxinos and Franklin (0.25 mm lateral, 0.50 mm posterior to bregma and at a depth of 5.10 mm). Mice were secured in a David Kopf stereotaxic frame (Kopf Instruments, Model 900). To reveal the skull, a midline incision in the scalp was made and two small holes were drilled above the location of the PVN. AAV-hSyn-DIO-hM3D(Gq)-mCitrine (150nl, 2.4 × 10^12^ GC/mL, Serotype:8; Addgene, Massachusetts, USA) was bilaterally injected into the PVN over the course of 10 min using a 5-μL Hamilton micro syringe (Esslab, Essex, UK). The needle was left in position for another 10 min before being withdrawn slowly over 1 min. Animals were given one week to recover, after which they were handled 20 min daily to be acclimatized to the serial tail-tip blood sampling procedure.

### DREADD activation of CRH neurons in the PVN

DREADD agonist compound 21 (C21, HB6124, Batch: E0877-5-1, Hello Bio Ltd., Bristol, UK) at the dose of 5 mg/kg per day was administered in drinking water 2 weeks after surgery and the treatment lasted for 3 weeks (16). Adult mice (∼25 g body weight) consume ∼5 mL of water per day (17). Accordingly, C21 dissolved in drinking water at 25mg/L results in 5 mg/kg (18), and the C21 water solution was changed daily. Water consumption was recorded daily. Body weight was measured every 3 days as normal experimental husbandry.

CRH-cre female mice injected with AAV-hSyn-DIO-hM3D(Gq)-mCitrine were randomly assigned to either a group with C21 in their drinking water (n = 16) or water alone (n = 13). As an additional control group, CRH-cre female mice with control virus (AAV-Ef1a-DIO-EYFP,150nl, 2.2 × 10^12^ GC/mL, Serotype:9; Addgene, Massachusetts, USA) injection received C21 in their drinking water (n = 8).

### Evaluation of estrous cycles

Vaginal smears were collected between 09:30 - 11:00 h daily 1 week before surgery. Only mice that showed regular estrous cycles were included. Vaginal smearing for experimental animals started from 3-day post-surgery until the end of 3-week C21 or water only control treatment. Smears were identified by microscopic examination of cell types and classified into 1 of 4 phases of the estrous cycle: proestrus, estrus, metestrus or diestrus. An intact estrous cycle was defined as positive classification of, at least, an estrus to diestrus to estrus transition (19). The phases of the estrous cycle were identified based on the prevalent cell type observed in the vaginal smears when examined under a light microscope (20).

### Blood sampling for LH pulses and LH surges

After surgery, mice were handled 20 min daily for 2 weeks before blood sample collection to minimize handling stress during blood sampling. Blood sample collection was initiated 7-10 days after C21 or control treatment onset, so that the animals were exposed to DREADDs activation of PVN CRH neurons for approximately 1 week before hormone measurement. The tail tip was excised using a sterile scalpel, and mice were left to habituate in a clean cage for 1 h before sampling for LH pulse measurement commenced. For LH pulse detection on diestrus or metestrus, the earliest opportunity to capture these 2 phases of the estrous cycle was randomly implemented, and a given mouse was only bled on one occasion, that is, either diestrus or metestrus. Blood samples (5-µL) were collected every 5 min for 2 h between 12:00-14:00 h, as previously described (21).

For LH surge detection on proestrus, again the earliest opportunity to capture this phase of the estous cycle was taken. Blood samples (5-µL) were collected between 15:30-21:00 h at 30 min intervals. At least 3 days elapsed between blood collection procedures for detection of LH pulsatility or surge to minimize stress. Samples were diluted in 45 μL of 0.2% bovine serum album in 0.05% PBS with Tween 20 and then were stored at -80°C before assay.

### Blood sampling for basal and restraint stress-induced corticosterone release

Blood sampling for basal CORT measurement was also initiated 7-10 days after C21 treatment onset and from the same cohort of animals used for LH measurements. On metestrus, (15-μL) blood samples were collected between 11:00 h and 12:00 h using the tail-tip bleeding procedure for basal CORT measurements. Animals were randomly selected from the CRH DREADDs activated and control groups. Moreover, the timing of the blood sample collection for basal CORT, and LH pulsatility described above, were randomly selected with typically 3-4 days between blood collection procedures to minimize stress. On a separate occasion, and after all other blood sampling for basal CORT and LH pulse or LH surge detection were complete, blood samples were collected between 11:00 h and 15:00 h for CORT measurement in response to restraint stress in metestrous mice. Blood samples (15-μL) were taken 30 min and 0 min before restraint onset, and 15, 30, 60 min after restraint onset. Blood samples were stored in tubes containing 5 μL heparinized saline (50 IU/mL). Blood samples were centrifuged at 3,000 RPM for 20 min at 4°C. Plasma was stored at -20°C before assay.

### LH and corticosterone measurement

LH blood samples were processed with a LH ELISA assay, as reported previously (22). The inter-assay and intra-assay variations were 10.7% and 4.9%, respectively. The assay sensitivity was 0.0015 ng/mL. Mouse LH standard (mLH; AFP-5306A, NIDDK-NHPP, USA), coating antibody (RRID: AB_2665514, monoclonal anti-bovine LH beta subunit antiserum, 518B7, University of California, CA, USA), anti-LH antibody (RRID: AB_2665533; National Hormone & Peptide Program, CA, USA) and a secondary antibody (RRID: AB_772206, GE Healthcare, Chicago, Illinois, USA) were used. Enzyme immunoassay kit (sheep polyclonal antibody specific for corticosterone, AB_2877626; DetectX W Enzyme Immunoassay Kit, K014; Arbor Assays, Michigan, USA) with an assay sensitivity of 20.9 pg/ml was used to determine the CORT concentrations in plasma samples.

### Validation of AAV injection

After experimental procedures, the mice were sacrificed with a lethal dose of ketamine and transcardially perfused by heparinized saline (5 U/ml) for 5 min, followed by 15 min of ice-cold 4% paraformaldehyde (PFA) (phosphate buffer 4% paraformaldehyde, pH 7.0) using a pump (Minipuls,156 Gilson, Villiers Le Bel, France). The brains were collected and post-fixed sequentially at 4°C in 15% sucrose in 4% PFA and 30% sucrose in phosphate buffer until they sank. They were then snap frozen on dry ice and stored in -80°C until processing. Brains were coronally sectioned (30 μm) between +0.5 mm and -2.7 mm from the bregma, and sections were mounted on microscope slides, air-dried, and covered with slips using Prolonged Antifade mounting medium (Molecular Probes, Inc. OR, USA). The position of injection was validated by Axioskop 2 Plus microscope equipped with axiovision 4.7 (Zeiss). The co-localization of red fluorescence (CRH-cre-tdTomato) and mCitrine fluorescence showed virus with successful DREADD(Gq) expression in PVN. Only the results of mice with correct viral infection in the PVN were included in the analysis.

### Statistical analysis

LH pulses were verified using the Dynpeak algorithm in Scilab 5.5.2 programme (23). LH pulse frequency was calculated. A level of LH higher than 2 ng/ml in intact proestrus mice was considered as a LH surge (24). Statistical significance was tested using a Mann-Whitney test unless otherwise stated. Data was represented as mean ± SEM and p< 0.05 was considered significant.

### Mathematical model and computer simulations

Our mathematical model is a phenomenological account of rhythmic activity within the HPA and HPG axes represented by a system of coupled oscillators. Accordingly, the model consists of a set of ordinary differential equations describing the changes in oscillatory properties resulting from physiological interactions within and between oscillators. For convenience, the model equations are reproduced below from Zavala et al. (15):


$$\frac{d}{dt}{\text{φ}}_{H}={\text{ω}}_{H_{0}}$$

$$\frac{d}{dt}{\text{φ}}_{C}={\text{ω}}_{C_{0}}-\text{α}s\left({\text{φ}}_{H}\right)$$

$$\frac{d}{dt}A_{C}=f_{H}\left({\text{φ}}_{H}\right)\frac{A_{E}^{n}}{A_{E}^{n}+K_{E}^{n}}-A_{C}+s\left({\text{φ}}_{H}\right)$$

$$\frac{d}{dt}{\text{φ}}_{PG}={\text{ω}}_{PG}$$

$$\frac{d}{dt}{\text{ω}}_{PG}={\text{ω}}_{PG_{m}}f_{K}\left(N,D,\tilde{C}\right)-{\text{ω}}_{PG}$$

$$\frac{d}{dt}{\text{φ}}_{E}={\text{ω}}_{E_{m}}f_{PG}\left({\text{ω}}_{PG}\right)$$

$$\frac{d}{dt}A_{E}=\text{ε}+\text{β}f_{E}\left({\text{φ}}_{E}\right)-A_{E}$$

where the phase, frequency and amplitude of the *i*-th oscillator are denoted by $\varphi_{i}$ , $\omega_{i}$ and , respectively, and *H*, *C*, *PG* and *E* denote the hypothalamic circadian drive, the CORT rhythm, the pulse generator and the estrous rhythm, respectively.

Computer simulations of the mathematical model developed by Zavala et al. (15) were performed in MATLAB R2022b using the ode45 numerical integrator. The model structure and parameter values remained the same, except for the frequency of the estradiol oscillator which was re-calibrated from 3 to 3.5 days to reflect the estrous cycle periodicity observed in control mice. C21 effects were assumed to occur at the level of the hypothalamus, affecting the dynamics of both the HPG and HPA axes. The C21 input was modeled as a square-wave function, with a duration equivalent to that of the experimental conditions (3 weeks of C21 in the drinking water) and 50% amplitude with respect to that of a typical acute stressor.

## Results

### Daily water intake and body weight

**Figure 1:**
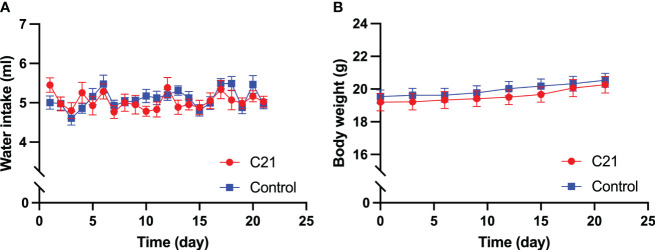
Effect of chronic chemogenetic activation (C21 in drinking water for 21 days) of PVN CRH neurons on daily water intake and body weight. Daily water intake **(A)** body weight **(B)** for the C21 and combined control groups respectively. Data are presented as mean ± SEM.

No significant difference was found in daily water intake (**Figure 1A**) and body weight (**Figure 1B**), that was measured every three days, between the C21 treatment (n=14) and combined control group (n = 18, water only, n = 11; control virus + C21, n = 7; 2‐way ANOVA with Sidak’s *post hoc* test for multiple comparisons).

### Validation of AAV injection in PVN

**Figure 2:**
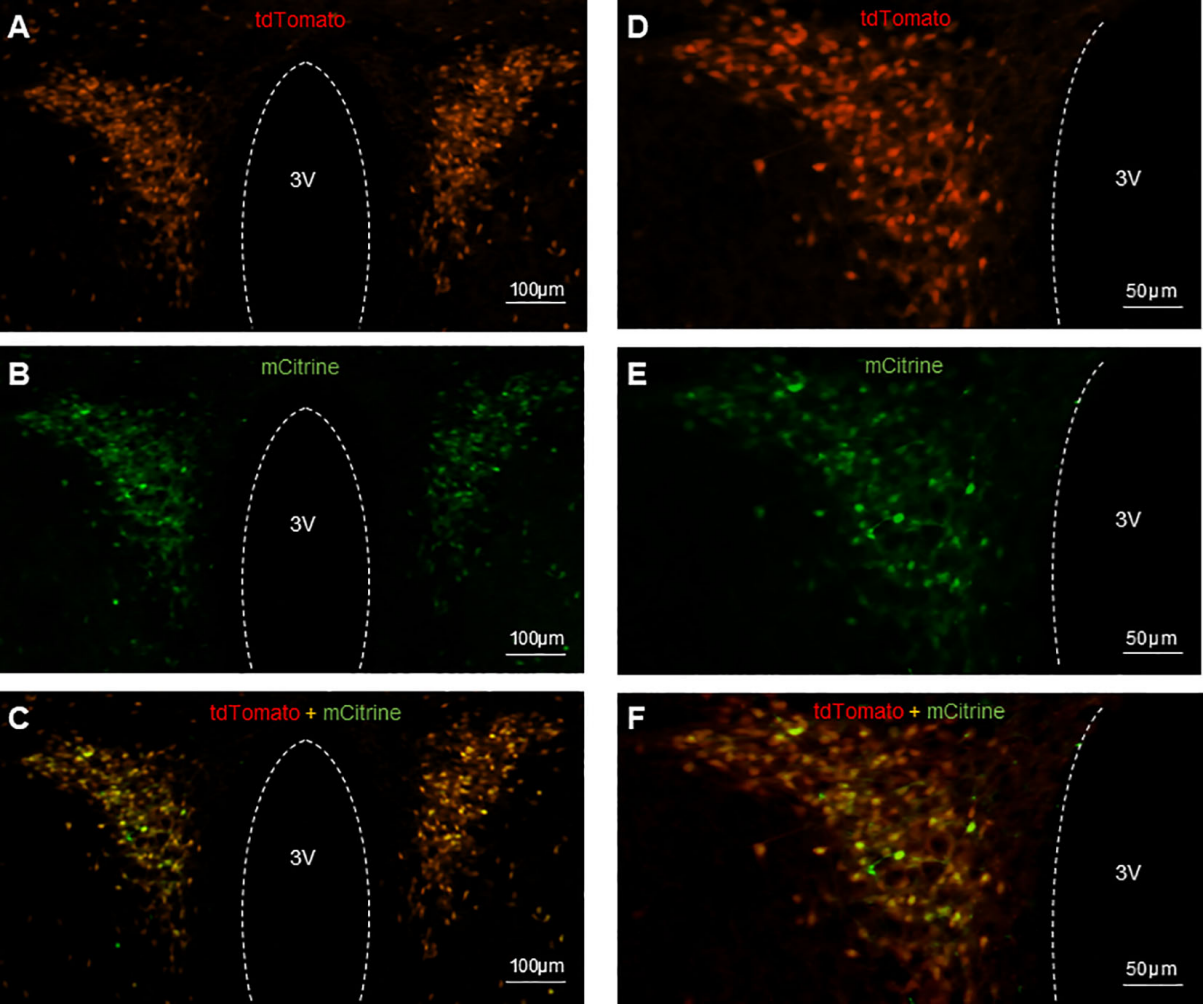
Expression of AAV-hSyn-DIO-hM3D(Gq)-mCitrine in PVN CRH neurons in CRH-Cre-tdTomato female mice. Representative examples of CRH neurons expressing tdTomato fluorescence **(A, D)**, hM3D labeled with mCitrine fluorescence **(B, E)** and colocalization of tdTomato and mCitrine in CRH neurons **(C, F)**. 3V, third ventricle.

The CRH neurons in CRH-cre-tdTomato mice were tagged with red fluorescence. The AAV-hSyn-DIO-hM3D(Gq)-mCitrine viral construct injected into the PVN allows the infected CRH neurons expressing green fluorescence to be visualized microscopically. Histology images obtained from coronal sectioning of the mice brains showed CRH neurons expressing tdTomato (**Figures 2A, D**), CRH neurons infected with mCitrine expressing virus (**Figures 2B, E**), and colocalization of tdTomato and mCitrine in CRH neurons (**Figures 2C, F**). Twenty-five out of 29 CRH-Cre female mice had successful located stereotaxic bilateral injection of hM3D(Gq)-mCitrine viral construct in the PVN. The mean ± SEM number of tdTomato CRH neurons in the PVN was 137.5 ± 4.74 per section, with 77.96 ± 3.94% cells coexpressing hM3D(Gq)-mCitrine. The off-target transduction of the hM3D(Gq)-mCitrine is 2.38 ± 0.46%, and no CRH neurons in other hypothalamic areas have been found infected. All 8 mice had successful bilateral injections of EYFP control virus in the PVN.

### The effect of PVN CRH neuron activation on estrous cycle in female mice

**Figure 3:**
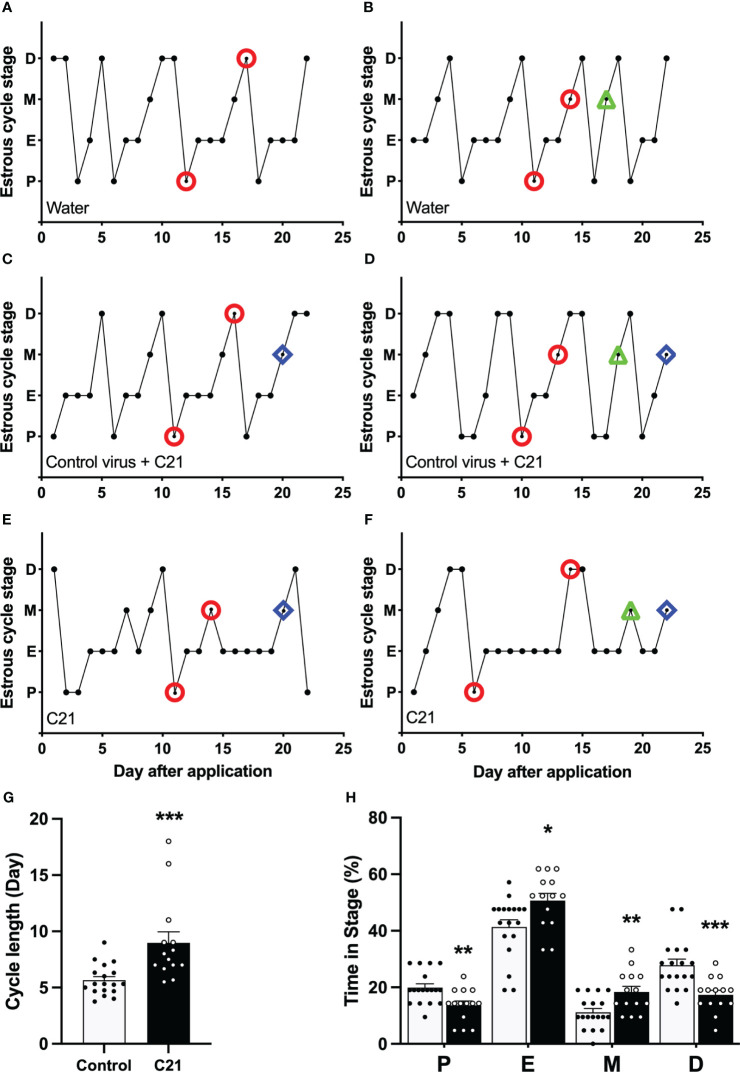
Chronic chemogenetic activation (C21 in drinking water for 21 days) of PVN CRH neurons disrupts the estrous cycles in mice. Representative examples of estrous cycles in the control water group **(A, B)**, control virus group **(C, D)**, and C21 group **(E, F)**. **(G)** Estrous cycle length in the C21 group and control animals combined (control water and control virus groups). **(H)** The percentage of time spent in each estrous stage in the C21 (black bar) and control group (white bar). P, proestrus; E, estrus; M, metestrus; D, diestrus. Representative examples of the time points for blood sample collection during course of study (○ = LH pulse on D and M or LH surge on P; △ = basal CORT on M and ◇ = CORT response to restraint on M). Data are presented as mean ± SEM. *P<0.05; **P<0.01; ***P<0.001 vs control.

Activation of PVN CRH neurons by C21 extended the estrous cycle length in female mice (**Figure 3**). Representative profiles of estrous cycles are shown as following: control water group (**Figures 3A, B**), control virus group receiving C21 (**Figures 3C, D**), C21 group (**Figures 3E, F**). Estrous cycle length in the C21 group (CRH-Cre female mice injected with AAV- hM3D(Gq) treated with C21; 8.98 ± 0.99 days, mean ± SEM, n = 14) was significantly lengthened compared with the combined control animal groups (n = 18, 5.66 ± 0.32 days, P=0.0002, Mann-Whitney test) (water only, n = 11, 5.44 ± 0.48 days; control virus + C21, n = 7, 5.99 ± 0.36 days: water vs control virus + C21, P>0.05, Mann-Whitney test) (**Figure 3G**). The percentage of time spent in proestrus and diestrus was significantly decreased in the C21 group (13.6% ± 1.6% in proestrus, 17.3% ± 1.6% in diestrus, mean ± SEM; n = 14 per stage) compared with the combined control animals (n = 18, 19.9% ± 1.3% in proestrus, P=0.0067, Mann-Whitney test, [water only, n = 11, 19.5% ± 2.0% vs control virus + C21, n = 7, 20.4% ± 1.7%, P>0.05, Mann-Whitney test]; 27.8% ± 2.1% in diestrus, P=0.0004 [water only, n = 11, 29.4% ± 3.0% vs control virus + C21, n = 7, 25.2% ± 2.7%, P>0.05, Mann-Whitney test]). In contrast, the percentage of time in estrus and metestrus were significantly increased in the C21 group (50.7% ± 2.6% in estrus, 18.4% ± 2.0% in metestrus; n = 14 per stage) compared with the combined control animals (n=18, 41.3% ± 2.6% in estrus, P=0.0133, Mann-Whitney test, [water only, n = 11, 39.4% ± 3.2% vs control virus + C21, n = 7, 44.2% ± 4.5%, P>0.05, Mann-Whitney test]; 11.1% ± 1.3% in metestrus, P=0.0067, Mann-Whitney test, [water only, n = 11, 11.7% ± 1.6% vs control virus + C21, n = 7, 10.2% ± 2.4%, P>0.05, Mann-Whitney test]); (**Figure 3H**).

### The effect of PVN CRH neuron activation on LH pulse frequency in diestrous and metestrous mice

**Figure 4:**
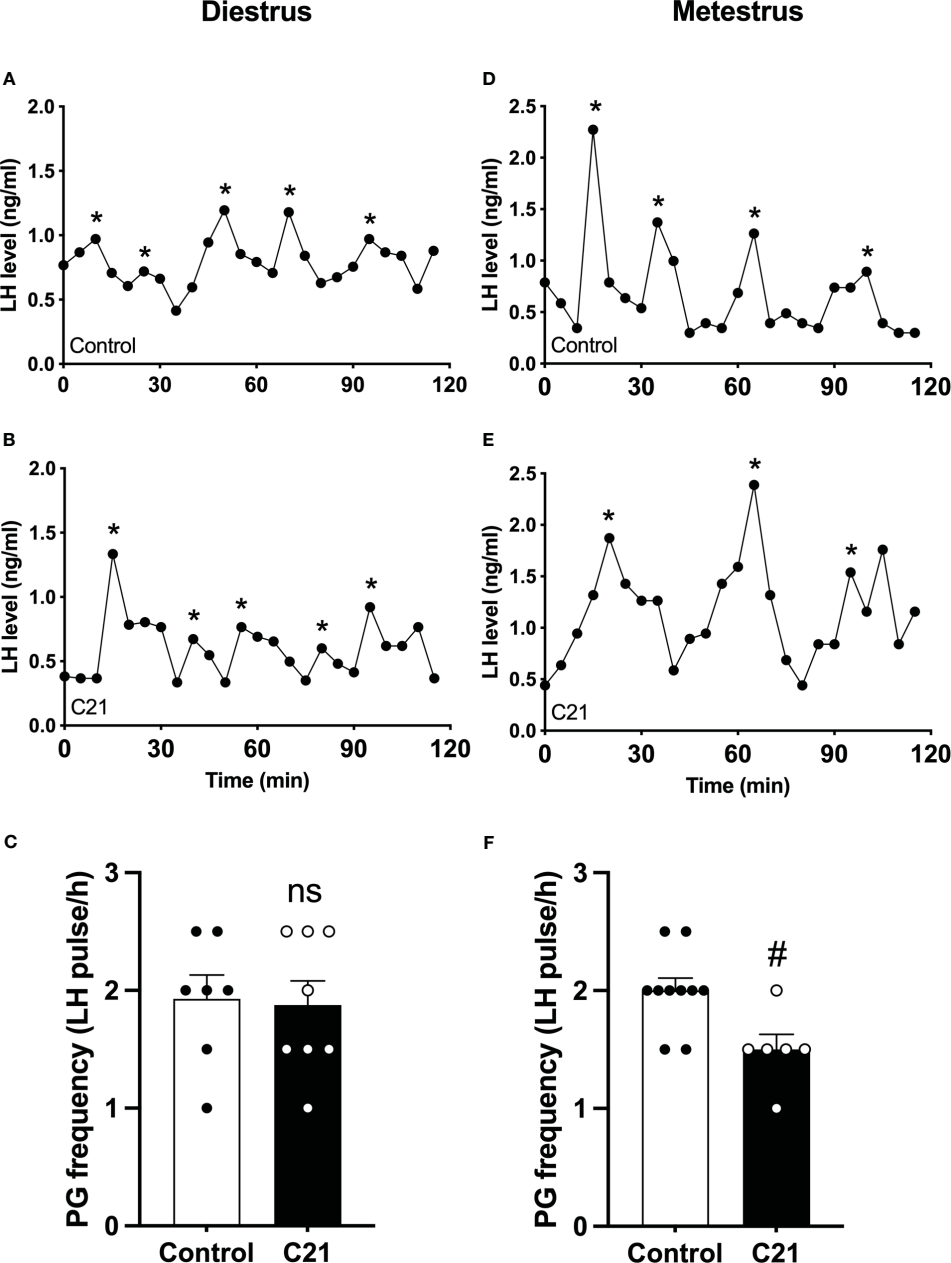
Chronic chemogenetic activation (C21 in drinking water for 21 days) of PVN CRH neurons decreased LH pulse frequency during metestrus in female mice. Representative LH pulse profiles of the control **(A, D)** and the C21 **(B, E)** group in diestrus and metestrus, respectively. Summary of GnRH pulse generator (PG) frequency (LH pulse/h) in the control and C21 groups in diestrus **(C)** and metestrus **(F)**. LH pulses detected with the DynPeak algorithm are indicated by an asterisk. Data are presented as mean ± SEM. **^#^** P<0.05 vs control. The "ns" means not significant.

The GnRH pulse generator frequency (LH pulse/h) in the C21 treated group (1.88 ± 0.21, mean ± SEM, n = 8) is comparable with the combined control group (1.93 ± 0.20, n = 7, P>0.05, Mann-Whitney test) (water only, n = 4; control virus + C21, n = 3) in diestrous mice (**Figure 4C**). However, in metestrus, the C21 group shows a significantly lower pulse generator frequency (1.50 ± 0.13, n=6) compared with the control group (2.00 ± 0.11, n = 10, P=0.0184, Mann-Whitney test) (water only, n = 5; control virus + C21, n = 5) (**Figure 4F**). Representative profiles of pulsatile LH secretion in diestrous animals drinking water only as a control (**Figure 4A**) or C21 (**Figure 4B**), and metestrous animals drinking water (**Figure 4D**) or C21 (**Figure 4E**). LH pulses detected with Dynpeak are indicated by an asterisk.

### The effect of PVN CRH neuron activation on LH surge in proestrous mice

**Figure 5:**
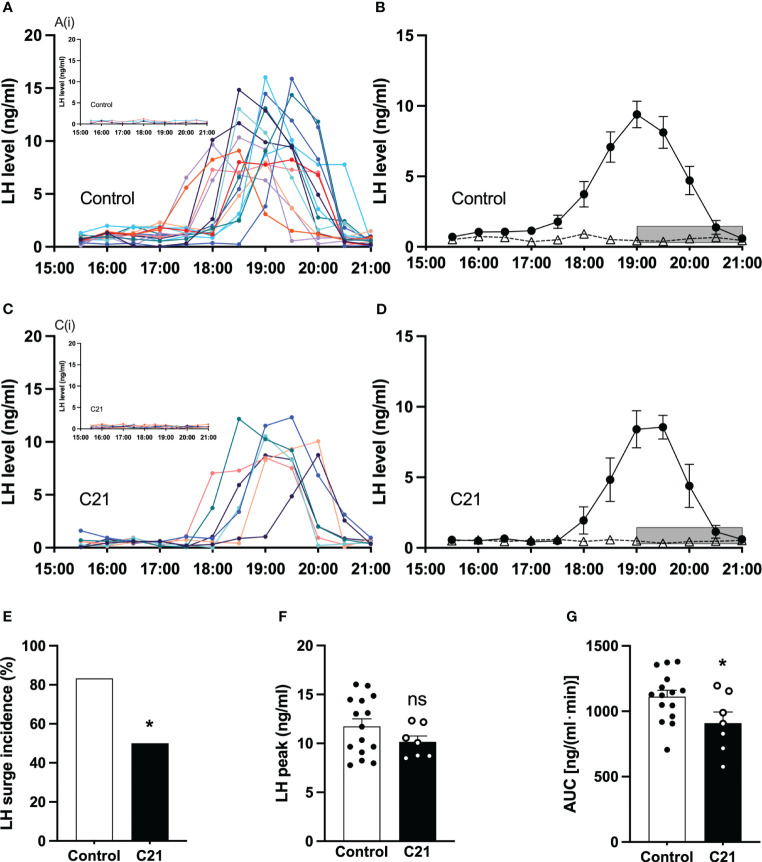
Chronic chemogenetic activation (C21 in drinking water for 21 days) of PVN CRH neuron activation decreased the incidence of proestrous LH surges and their area under the curve (AUC) in mice. The individual LH surge profiles are illustrated for the control group **(A)** and C21 group **(C)**; the inset figures [A(i) and C(i)] show the individuals without LH surge in both groups, respectively. Mean (± SEM) circulating levels of LH in proestrus female mice with spontaneous LH surges (closed circle) or absence of LH surges (open triangle) are shown for the control group **(B)** and C21 group **(D)** respectively. Grey bar represents lights off at 19:00 h. The LH surge incidence **(E)**, LH surge peak **(F)** and area under curve (17:00 – 21:00 h) of LH levels **(G)** in control and C21 group. Data are presented as mean ± SEM. *P<0.05 vs control. The "ns" means not significant.

All blood collection procedures for detection of LH surges coincided with a single proestrous event and were followed by transition to estrus the next day. The individual spontaneous LH surge profiles for proestrous mice in the control group and the C21 treatment group are shown in **Figures 5A, C** respectively. The insert **Figure 5**Ai (water only, n = 2; control virus + C21, n = 1) and **Figure 5**Ci (n = 7) show individual LH profiles without LH surges in both groups respectively. Additionally, we present the mean ± SEM circulating levels of LH in proestrous female mice with or without spontaneous LH surges is the control group (**Figure 5B**: combined control group, n = 15; composed of water only, n = 8 and control virus + C21, n = 7) and C21 group (**Figure 5D**, n = 7). 7 out of 14 mice in the C21 treatment group showed LH surges on proestrus. 15 out of 18 mice in the control groups showed LH surges on proestrus. The LH surge incidence of the C21 group (50%) is significantly lower than that of control group (83.3%, P=0.044, Chi-square test) (**Figure 5E**). There was no relationship between a positive or negative LH surge outcome on proestrus and the number of sequential days of estrus that followed irrespective of treatment group. There is no significant difference in the LH surge peak between C21 and control group (P>0.05, unpaired t test) (**Figure 5F**). The area under the curve of LH levels (AUC, 17:00-21:00 h) in the C21 group (n=7, 909.3 ± 84.98 ng/(ml·min), mean ± SEM) is significantly lower than that in control group (n=15, 1111.0 ± 48.83 ng/(ml·min), P=0.0396, unpaired t test) (**Figure 5G**).

### The effect of PVN CRH neuron activation on CORT release

**Figure 6:**
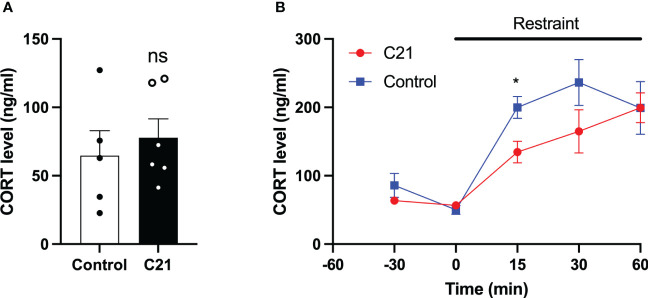
Effect of chronic chemogenetic activation (C21 in drinking water for 21 days) of PVN CRH neuron on basal and stress induced levels of corticosterone (CORT) in metestrous mice. **(A)** Summary of basal CORT levels measured by single time point determination between 11:00 and 12:00 h in control and C21 groups. **(B)** Summary of the CORT response to acute restraint stress (1 h) determined between 11:00 – 15:00 h in the control and C21 group. Data are presented as mean ± SEM. *P<0.05 vs C21 at same time point. The "ns" means not significant.

Single blood samples collected in the morning between 11:00 h and 12:00 h showed that the basal CORT levels in C21 treatment group (77.79 ± 13.77 ng/ml, mean ± SEM, n=6) and combined control group (64.63 ± 18.30 ng/ml, mean ± SEM, n=5, composed of water only, n = 2 and control virus + C21, n = 3) are comparable on metestrus (**Figure 6A**). The CORT response to restraint stress (1 h) was determined between 11:00 h – 15:00 h on metestrus. CORT levels were similar at 30 min and 0 min before stress onset in the C21 and control groups but increased during restraint (**Figure 6B**). However, the combined control mice (199.8 ± 16.08 ng/ml, n = 6, composed of water only, n = 2 and control virus + C21, n = 4) showed a greater increase in CORT levels at 15 min after restraint onset compared to the C21 group (134.7 ± 15.91 ng/ml, mean ± SEM, n = 6; P< 0.05, 2-way repeated measures ANOVA with a Bonferroni *post-hoc* analyses).

### Computational simulation of C21 effects

**Figure 7:**
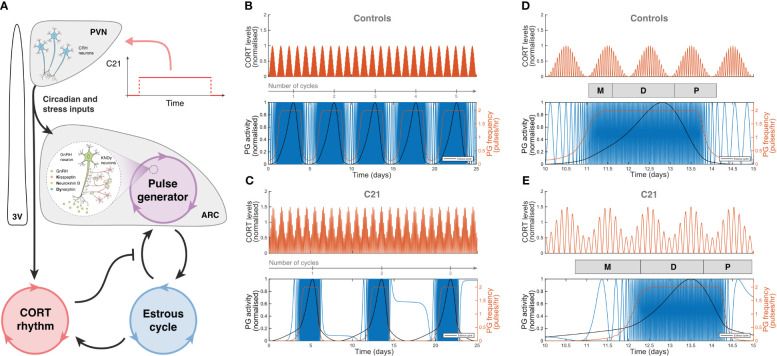
Computer simulations of HPA and HPG axes rhythmic activity generated by our mathematical model. **(A)** Graphic representation of the mathematical model introduced in Zavala et al. (15), including a C21 input function upstream of the HPA and HPG axes. Simulated CORT and pulse generator rhythmic activity in **(B)** control mice, and **(C)** C21-treated mice illustrate the lengthening of the estrous cycle. A 5-day observation window of **(D)** control mice and **(E)** C21-treated mice show the relative change in the duration of the estrous cycle phases (**Table 1**). In **(B–E)**, the red line in the upper panel represents the oscillatory activity of CORT, normalized to the control case. The blue, red and black lines in the bottom panels respectively account for the hypothalamic pulse generator activity (experimentally reported by LH measurements), the pulse generator frequency (in pulses per hour), and the estrous cycle oscillator output.

The effects of C21 were simulated computationally using the mathematical model previously introduced in Zavala et al. (15). Our simulations assumed sustained hypothalamic activation over the period of time the animals had C21 in their drinking water (**Figure 7A**). The model was first re-calibrated to reproduce the estrous cycle periodicity in control mice (**Figure 7B**). Subsequent simulation of C21 effects predicted a lengthening of the estrous cycle, an increased amplitude of CORT oscillations (which averaged over time would amount to an increased baseline), and a reduction of the PG high-frequency region width (**Figure 7C**) compared to controls. The number of complete estrous cycles predicted by the model over a 21-day period was consistent with the experimental observations in **Figures 3A–F**. A 5-day long detail of these simulations allow us to map the length of each estrous cycle phase in the control (**Figure 7D**) and C21 groups (**Figure 7E**). Importantly, despite the estrous cycle being longer in the C21 group, the 5-day observation window comparison illustrates the shrinking of the PG high-frequency region. This reveals some interesting observations, for instance, while the duration of the diestrus phase measured in days is approximately the same in the C21 (1.48 days) vs the control group (1.5 days), it constitutes a smaller percentage of the total estrous cycle length in C21 (16.5%) than in controls (26.5%). In contrast, the duration of the metestrous phase in the C21 group (1.57 days, 17.5% of the “elongated” estrous cycle length) is by any account longer than the control group (0.6 days, 10.6% of the “normal” estrous cycle length). The duration of all estrous cycle phases, estimated in days and as a percentage of the estrous cycle length is shown in **Table 1**. Lastly, it is worth noting that the predicted pulse generator frequency in metestrous (~0.5 pulses/hr) (**Figure 7E**) was lower than the experimental observation (~1.5 pulses/hr) (**Figure 4F**). This is likely related to the intrinsic limitations of using a phenomenological model –where the state variables are not molecular concentrations but oscillator properties. However, a more detailed mechanistic approach would be constrained by the limited availability of time series data typically needed to calibrate such models and hence is beyond the scope of the present study.

**Table 1:** Estimated length (<5% error) of estrous cycle phases in days and as a percentage of the total estrous cycle length in C21 (8.98 ± 0.99 days) and control groups (5.66 ± 0.32 days), respectively.

Cycle phase	Controls		C21	
	Days	Percentage	Days	Percentage
Estrus (E)	2.23	39.4	4.35	48
Metestrus (M)	0.6	10.6	1.57	17.5
Diestrus (D)	1.5	26.5	1.48	16.5
Proestrus (P)	1.07	19	1.17	13

## Discussion

In the present study the estrous cycle was prolonged in response to chronic chemogenetic activation of PVN CRH neurons in mice. Specifically, there was an increase in the time spent in estrus and metestrus and a decrease in diestrus and proestrus. Although LH pulse frequency was not affected in the diestrous phase, there was a decrease during metestrus in response to CRH neuronal activation. Additionally, C21 treatment led to a significant reduction in the incidence of LH surges from a normal 83% to 50% and overall surge AUC was reduced. Although basal CORT levels in the late morning were unaffected by chronic PVN CRH neuron stimulation, the CORT response to acute restraint stress in late morning-early afternoon was attenuated.

Zavala et al. suggested that the HPA and HPG axes act as coupled oscillators (15). The model predicted that hyperactivation of the HPA axis leading to hypercortisolism can extend the overall length of rodent estrous cycles. More specifically, a lengthening of the estrous and metestrous stages, but shortening of the diestrous and proestrous stages (relative to the cycle length). Using CORT implantable pellets in female mice, a previous study partly confirmed these predictions by showing a lengthening of the estrous cycle (25). However, these animals showed almost constant diestrus with shortened estrus and proestrus, which is inconsistent with our mathematical model prediction (15), indicating that the effects of HPA axis hyperactivation on the estrous cycle may be more complex than initially thought. This discrepancy may be due, in part, to different methods of experimentally mimicking HPA axis hyperactivation. While Kellie Breen and colleagues (25) used implantable pellets to generate chronic high stress levels of CORT, the present study chronically activated the HPA axis at the PVN CRH neuron level using chemogenetics. Our data showed more subtle prolonged estrous cycles with decreased proestrous and diestrus, and increased estrous and metestrous stages, confirming our mathematical model predictions. This central HPA activation results in different effects on the individual phases of the estrous cycle than peripheral activation via CORT, suggesting that there may be different pathways through which the different levels of the HPA axis affect the estrous cycle. Additionally, the extended cycle length originating from increased estrus and metestrus could potentially mean that it takes a longer time for the GnRH pulse generator to increase frequency in chronically stressed mice, as the estrus phase is associated with a very low frequency of the GnRH pulse generator (26). Stress-induced HPA axis hyperactivation may have a greater impact on the post-ovulatory phase (estrus and metestrus) of the estrous cycle than the pre-ovulatory phase (diestrus and proestrus). However, the mechanism in which different cycle stages have distinct alteration still needs further investigation.

The mathematical model of Zavala et al. made an interesting prediction that GnRH pulse generator frequency remains robust in the presence of stressful perturbations on diestrous and early proestrous stage but is fragile toward stressors on estrous and metestrous stages (15). This is confirmed in the present study, where the pulsatile LH secretion is unaffected by chronic chemogenetic activation of PVN CRH neurons in diestrus but decreased in the metestrous phase. Acute chemogenetic (11) and optogenetic (12) stimulation of PVN CRH neurons dramatically suppresses pulsatile LH secretion in OVX and OVX + E_2_ female mice, respectively. Similarly, acute restraint stress suppresses pulsatile LH secretion in OVX mice (27). In OVX animals, the effect of the natural dynamics of the estrous cycle hormone milieu on stress responsivity in terms of LH pulsatility has not been adequately addressed. This compounds with the fact that chronic stress models are more ecologically relevant to our highly competitive modern society and the animal kingdom in general. The current investigation used, for the first time, a chronic stress intact female mouse model to confirm the robustness and vulnerability of GnRH pulse generator activity in diestrus and metestrus, respectively. According to the mathematical model, the robustness of the GnRH pulse generator to stress in diestrus might represent a protective mechanism to maintain follicular development in key stages close to ovulation. In contrast, it is conceivable that when the GnRH pulse generator frequency is lower in estrus, it is less robust and more susceptible to stress-induced perturbations (15). Ecologically, this plasticity to stress could potentially prevent unnecessary follicular development and maintain a more quiescent HPG axis until the stressful period has passed, thus conserving energy and resources. This might potentially be beneficial for avoiding unnecessary recruitment and activation of primordial follicles, maintaining the follicular reserve (28). The differential sensitivity of the GnRH pulse generator to stressful perturbations across the ovarian cycle could be argued as counterintuitive as continuous invariant sensitivity and reduced pulse generator frequency under stress would prevent follicle maturation and ovulation. Is it possible, however, that reproduction is so critical for survival that important brain systems evolved supplementary mechanisms to allow “escape”, where possible, and the robustness predicted and observed in the present study leading to an LH surge and ovulation is an expression of that evolutionary adaptation? Its expression may depend on the intensity, nature and duration of the stressor. However, the adaptive response of the GnRH pulse generator to stress is an interesting hypothesis and provides a useful framework for understanding the interplay between stress and reproductive function. Nevertheless, further research is needed to fully understand the mechanisms underlying this plasticity and how it impacts reproductive outcomes in different species and stress conditions. The PVN CRH neurons receive inputs from multiple brain areas conveying different homeostatic or stress modalities to activate the HPA axis. The diverse nature of the neurotransmitter and neuropeptide afferents coupled with heterosynaptic modulation of the CRH neuron activity (29) tempers the argument that chronic Gq-DREADDs activation of PVN CRH is not dissimilar to chronic stress activation of the CRH neurons and is therefore a limitation of the present study. Additionally, the model indicates that the extended estrus and metestrus are all characterized with a much lower GnRH pulse generator frequency than unstressed mice. A second-generation mechanistic (quantitative) model of GnRH/LH pulsatility should address these inaccuracies by correctly estimating the values of kinetic parameters after fitting its predictions to experimental data. However, the serial blood sampling method of determining frequency would have to significantly overcome current temporal limitations before such mechanistic models can be duly calibrated.

The LH surge is a critical event in the female reproductive cycle that triggers ovulation. Unlike the pulsatile secretion of LH, the LH surge is under the control of a different population of kisspeptin neuron in the AVPV (GnRH surge generator) (30–33). In the present study, chronic C21 treatment reduced the incidence of mice showing proestrus LH surge and attenuated the surge AUC. The former could potentially indicate fewer ovulatory episodes. The disruption of LH surge is in accordance with previous stress studies. Acute psychosocial stress (24) and chronic undernutrition (19) can reduce LH surge incidence in female mice. This indicates that the GnRH surge generator may not have the same robustness as the GnRH pulse generator around the ovulatory phase. It is conceivable that the LH surge is subject to hormonal feedback regulation, making it more susceptible to the influence of external factors. It has been reported that chronic cold stress blocks LH surge in the rats, moreover the levels of estradiol and progesterone in proestrus show a significant decrease level, which might fail to evoke the positive feedback effect on AVPV kisspeptin neurons (34).

In this study, the estrous cycle and LH release were disrupted after long-term chemogenetic stimulation in PVN CRH neurons, but the basal level of CORT did not increase. There are two potential reasons for this. Firstly, the secretion pattern alteration of CORT under chronic chemogenetic activation of CRH neurons might be time of day dependent. It has been reported that mice exposed to chronic variable stress (CVS) showed similar basal CORT levels in the morning (09:00 h - 11:00 h), but higher CORT concentration in the late afternoon (17:00 h) (35). In the present study, CORT blood samples were collected in the morning and therefore this potential diurnal component would have been missed, and such an elevated CORT secretion might disrupt reproductive function via inhibiting kisspeptin neurons in ARC and AVPV in female mice (25, 36). Since CORT is inherently dynamic, with near-hourly ultradian pulses in rodents, assessing baseline levels would require sampling over longer periods of time, and ideally over 24 h, which is a limitation of the current study. The diurnal difference in CORT levels in response to chronic stress might have other physiological sequalae. CORT is known to play a role in regulating the sleep-wake cycle, with higher levels of CORT promoting wakefulness and inhibiting sleep (37). Thus, an increase in CORT secretion in the active phase, may be adaptive in promoting wakefulness and maintaining the vigilance necessary to deal with potential threats in the environment. However, prolonged exposure to elevated CORT levels has been associated with several adverse health outcomes, including suppression of the immune system, increased susceptibility to infection, cardio-metabolic disease, cognitive decline in addition to impaired reproductive function (38). Thus, a blunted CORT response in the inactive phase may be more beneficial to the organism. Understanding the time-of-day dependent changes in CORT secretion under chronic stress can provide insight into the mechanisms underlying the negative health outcomes associated with chronic stress and may aid in the development of targeted interventions to mitigate these effects. Secondly, the HPA axis can undergo allostatic adaptation following chronic stimulation. Although the basal levels of CORT in both groups were comparable, our study found that the control group had a higher CORT increase in response to acute restraint stress compared to the C21 treated group. This contrast with a comparable CORT response to restraint stress in the morning (09:00 – 11:00 h) in CVS exposed mice (35). However, Handa and colleagues (35) did show that exposure to the acute restraint stress in the late afternoon (17:00 h) blunted the CORT response in CVS treated female mice. The mechanisms underlying these time-of-day differences in the CORT response to acute restraint stress in animals chronically exposed to stress remains to be established. Others have also shown that basal CORT levels did not change in CVS treated mice (4), or in chronic repeated restraint (39, 40), or repeated cat odor exposure (41) in the rat. These results indicate that a higher stimulation level is required for CORT secretion activity in the adrenal gland after prolonged activation of CRH neurons, or that the synthesis capacity may be compromised, leading to a decrease in secretion function. If so, how can we account for the reproductive disruptions without basal CORT alteration?

It may be because CRH activation directly impacts kisspeptin or GnRH neurons, modulating LH secretion. Double-labeling immunohistochemistry revealed that most kisspeptin neurons in the AVPV and ARC expressed CRH receptors and CRH projection fibers are found in these nuclei (42). CRH exhibits inhibitory effects on GnRH neuron firing activity *in vitro* via a CRH receptor type 2 mechanism in OXV E_2_ replaced mice (43). These data indicate that chronically activated CRH neurons in the PVN might be able to directly or indirectly impact kisspeptin or GnRH neurons over a long time without downstream HPA activity. Recently, we have shown that optical stimulation of PVN CRH neurons or PVN GABAergic projection terminals in the ARC suppressed LH pulses to a comparable degree in OVX E_2_-replaced female mice (12). Moreover, the PVN CRH neuron optical stimulation induced suppression of LH pulses was blocked by simultaneous optical inhibition of GABAergic neuron cell body in the PVN using intersectional vector strategies. Interestingly, the elevated CORT levels were augmented by this intervention compared with PVN CRH neuron optical stimulation alone. These findings suggest that PVN GABAergic signaling could potentially be the underlying mechanism responsible for the suppression of GnRH pulse generator activity induced by PVN CRH activation, independent of CORT (12).

In addition to the projection of PVN CRH neurons to the median eminence, they are known to project to several brain loci, including the lateral hypothalamic, amygdala, bed nucleus of the stria terminalis, periaqueductal gray, locus coeruleus and dorsal raphe (44, 45). The projection of PVN CRH neurons to the lateral hypothalamus is known to control stress coping behavior independent of their ability to release hormones (46). These findings indicate that PVN CRH neuron might respond to stress via direct synaptic function independent of neuroendocrine effects. Finally, CRH has been reported to increase and maintain a high expression level under long term stress. Repeated daily cat exposure (41), CVS (47–49) and repeated immobilization (50) up-regulated PVN mRNA CRH expression in the rat. It has also been demonstrated that PVN neurons remain active after long-term glucocorticoid negative feedback (51), and chronic stress may even increase the excitability of PVN CRH neurons via multiple mechanisms (52). Our mathematical modeling could also be extended to interrogate other physiological phenomena such as the mechanisms by which the PVN CRH neural system underlies the adverse outcomes of nutritional programming on cardiovascular disease (53). Another important consideration is the fact that drinking water behavior is not constant throughout the day. Whether this impacts the dynamic pattern of Gq activation of CRH neurons remains to be explored. While data on specific drinking events was not collected in the present study, the C21 input function used in the model could be modified to account for episodic drinking behavior should future studies require it.

In summary, the chronic activation of PVN CRH neurons using chemogenetics plays a role in mimicking stress-induced disruption of reproductive function. The estrous cycle in mice was prolonged under these chronic stress conditions, with extended estrus, metestrus and shortened diestrus and proestrus. The LH pulse frequency remained robust in diestrus but was decreased in metestrus. Additionally, the preovulatory LH surge was disrupted in proestrus. These findings confirmed the predictions of a first-generation mathematical model accounting for the dynamic control of stress and fertility (15).

## Data availability statement

The raw data supporting the conclusions of this article will be made available by the authors, without undue reservation.

## Ethics statement

The animal study was reviewed and approved by the Animal Welfare and Ethical Review Body Committee at King’s College London.

## Author contributions

JY: Conceptualization, Data curation, Formal Analysis, Investigation, Methodology, Validation, Writing – original draft, Writing – review & editing. X-FL: Conceptualization, Funding acquisition, Investigation, Methodology, Project administration, Writing – original draft, Writing – review & editing. KT-A: Conceptualization, Methodology, Writing – review & editing. EZ: Conceptualization, Data curation, Formal Analysis, Investigation, Methodology, Software, Validation, Writing – original draft, Writing – review & editing. KO’B: Conceptualization, Funding acquisition, Investigation, Methodology, Project administration, Writing – original draft, Writing – review & editing.

## References

[1] McCosh RB O'Bryne KT Karsch FJ Breen KM. Regulation of the gonadotropin-releasing hormone neuron during stress. J Neuroendocrinol (2022) 34(5):e13098. doi: 10.1111/jne.13098 35128742 PMC9232848

[2] Fergani C Saifullizam A Routly J Smith R Dobson H. Estrous behavior, luteinizing hormone and estradiol profiles of intact ewes treated with insulin or endotoxin. Physiol Behav (2012) 105(3):757–65. doi: 10.1016/j.physbeh.2011.09.025 22015330

[3] Breen KM Billings HJ Debus N Karsch FJ. Endotoxin inhibits the surge secretion of gonadotropin-releasing hormone via a prostaglandin-independent pathway. Endocrinology (2004) 145(1):221–7. doi: 10.1210/en.2003-1102 14551234

[4] Nair BB Khant Aung Z Porteous R Prescott M Glendining KA Jenkins DE. Impact of chronic variable stress on neuroendocrine hypothalamus and pituitary in male and female C57BL/6J mice. J Neuroendocrinol (2021) 33(5):e12972. doi: 10.1111/jne.12972 33896057

[5] Li XF Knox AMI O'Byrne KT. Corticotrophin-releasing factor and stress-induced inhibition of the gonadotrophin-releasing hormone pulse generator in the female. Brain Res Bull (2010) 1364:153–63. doi: 10.1016/j.brainres.2010.08.036 20727865

[6] Rosenfeld D Senko AW Moon J Yick I Varnavides G Gregureć D. Transgene-free remote magnetothermal regulation of adrenal hormones. Sci Adv (2020) 6(15):eaaz3734–eaaz3734. doi: 10.1126/sciadv.aaz3734 32300655 PMC7148104

[7] Arnett MG Muglia LM Laryea G Muglia LJ. Genetic approaches to hypothalamic-pituitary-adrenal axis regulation. Neuropsychopharmacology (2016) 41(1):245–60. doi: 10.1038/npp.2015.215 PMC4677126 26189452

[8] Kakizawa K Watanabe M Mutoh H Okawa Y Yamashita M Yanagawa Y. A novel GABA-mediated corticotropin-releasing hormone secretory mechanism in the median eminence. Sci Adv (2016) 2(8):e1501723–e1501723. doi: 10.1126/sciadv.1501723 27540587 PMC4988769

[9] McCullough KM Chatzinakos C Hartmann J Missig G Neve RL Fenster RJ. Genome-wide translational profiling of amygdala Crh-expressing neurons reveals role for CREB in fear extinction learning. Nat Commun (2020) 11(1):5180–0. doi: 10.1038/s41467-020-18985-6 PMC7560654 33057013

[10] Rivest S River C. Influence of the paraventricular nucleus of the hypothalamus in the alteration of neuroendocrine functions induced by intermittent footshock or interleukin. Endocrinology (1991) 129(4):2049–57. doi: 10.1210/endo-129-4-2049 1655391

[11] Yip SH Liu X Hessler S Cheong I Porteous R Herbison AE. Indirect suppression of pulsatile LH secretion by CRH neurons in the female mouse. Endocrinology (2021) 162(3):1–17. doi: 10.1210/endocr/bqaa237 33543235

[12] McIntyre C Li XF Ivanova D Wang J O'Byrne KT. Hypothalamic PVN CRH neurons signal through PVN GABA neurons to suppress GnRH pulse generator frequency in female mice. Endocrinology (2023) 164(6):1–17. doi: 10.1210/endocr/bqad075 PMC10234117 37246581

[13] Seale J Wood S Atkinson H Harbuz M Lightman S. Gonadal steroid replacement reverses gonadectomy-induced changes in the corticosterone pulse profile and stress-induced hypothalamic-pituitary-adrenal axis activity of male and female rats. J Neuroendocrinol (2004) 16(12):989–98. doi: 10.1111/j.1365-2826.2004.01258.x 15667454

[14] Zavala E Wedgwood KCA Voliotis M Tabak J Spiga F Lightman SL. Mathematical modelling of endocrine systems. Trends Endocrinol Metab (2019) 30(4):244–57. doi: 10.1016/j.tem.2019.01.008 PMC6425086 30799185

[15] Zavala E Voliotis M Zerenner T Tabak J Walker JJ Li XF. Dynamic hormone control of stress and fertility. Front Physiol (2020) 11:598845. doi: 10.3389/fphys.2020.598845 33329048 PMC7718016

[16] Thompson KJ Khajehali E Bradley SJ Navarrete JS Huang XP Slocum S. DREADD agonist 21 is an effective agonist for muscarinic-based DREADDs *in vitro* and *in vivo* . ACS Pharmacol Trans Sci (2018) 1(1):61–72. doi: 10.1021/acsptsci.8b00012 PMC6407913 30868140

[17] Guo W Wan X Ma L Zhang J Hashimoto K. Abnormalities in the composition of the gut microbiota in mice after repeated administration of DREADD ligands. Brain Res Bull (2021) 173:66–73. doi: 10.1016/j.brainresbull.2021.05.012 34004259

[18] Oyama K Hori Y Nagai Y Miyakawa N Mimura K Hirabayashi T. Chronic behavioral manipulation via orally delivered chemogenetic actuator in macaques. J Neurosci (2022) 42(12):2552–61. doi: 10.1523/JNEUROSCI.1657-21.2021 PMC8944228 35110390

[19] Kreisman MJ Tadrousse KS McCosh RB Breen KM. Neuroendocrine basis for disrupted ovarian cyclicity in female mice during chronic undernutrition. Endocrinology (2021) 162(8):bqab103. doi: 10.1210/endocr/bqab103 34037744 PMC8272537

[20] Cora MC Kooistra L Travlos G. Vaginal cytology of the laboratory rat and mouse: review and criteria for the staging of the estrous cycle using stained vaginal smears. Toxicol Pathol (2015) 43(6):776–93. doi: 10.1177/0192623315570339 PMC11504324 25739587

[21] McIntyre C Li XF de Burgh R Ivanova D Lass G O'Byrne KT. GABA signaling in the posterodorsal medial amygdala mediates stress-induced suppression of LH pulsatility in female mice. Endocrinology (2022) 164(1):1–11. doi: 10.1210/endocr/bqac197 PMC9757692 36453253

[22] Steyn FJ Wan Y Clarkson J Veldhuis JD Herbison AE Chen C. Development of a methodology for and assessment of pulsatile luteinizing hormone secretion in juvenile and adult male mice. Endocrinology (2013) 154(12):4939–45. doi: 10.1210/en.2013-1502 PMC5398599 24092638

[23] Vidal A Zhang Q Medigue C Clement F. DynPeak: an algorithm for pulse detection and frequency analysis in hormonal time series. PloS One (2012) 7(7):e39001. doi: 10.1371/journal.pone.0039001 22802933 PMC3389032

[24] Wagenmaker ER Moenter SM. Exposure to acute psychosocial stress disrupts the luteinizing hormone surge independent of estrous cycle alterations in female mice. Endocrinology (2017) 158(8):2593–602. doi: 10.1210/en.2017-00341 PMC5551545 28549157

[25] Luo E Stephens SB Chaing S Munaganuru N Kauffman AS Breen KM. Corticosterone blocks ovarian cyclicity and the LH surge via decreased kisspeptin neuron activation in female mice. Endocrinology (2016) 157(3):1187–99. doi: 10.1210/en.2015-1711 PMC4769373 26697722

[26] McQuillan HJ Han SY Cheong I Herbison AE. GnRH pulse generator activity across the estrous cycle of female mice. Endocrinology (2019) 160(6):1480–91. doi: 10.1210/en.2019-00193 31083714

[27] Yang JA Song CI Hughes JK Kreisman MJ Parra RA Haisenleder DJ. Acute psychosocial stress inhibits LH pulsatility and kiss1 neuronal activation in female mice. Endocrinology (2017) 158(11):3716–23. doi: 10.1210/en.2017-00301 PMC5695836 28973125

[28] Fischer-Holzhausen S Roblitz S. Hormonal regulation of ovarian follicle growth in humans: Model-based exploration of cycle variability and parameter sensitivities. J Theor Biol (2022) 547:111150. doi: 10.1016/j.jtbi.2022.111150 35568223

[29] Sunstrum JK Inoue W. Heterosynaptic modulation in the paraventricular nucleus of the hypothalamus. Neuropharmacology (2019) 154:87–95. doi: 10.1016/j.neuropharm.2018.11.004 30408488

[30] Uenoyama Y Inoue N Nakamura S Tsukamura H. Kisspeptin neurons and estrogen-estrogen receptor alpha signaling: unraveling the mystery of steroid feedback system regulating mammalian reproduction. Int J Mol Sci (2021) 22(17):1–16. doi: 10.3390/ijms22179229 PMC8430864 34502135

[31] Goodman RL Herbison AE Lehman MN Navarro VM. Neuroendocrine control of gonadotropin-releasing hormone: Pulsatile and surge modes of secretion. J Neuroendocrinol (2022) 34(5):e13094. doi: 10.1111/jne.13094 35107859 PMC9948945

[32] Sobrino V Avendano MS Perdices-Lopez C Jimenez-Puyer M Tena-Sempere M. Kisspeptins and the neuroendocrine control of reproduction: Recent progress and new frontiers in kisspeptin research. Front Neuroendocrinol (2022) 65:100977. doi: 10.1016/j.yfrne.2021.100977 34999056

[33] Starrett JR Moenter SM. Hypothalamic kisspeptin neurons as potential mediators of estradiol negative and positive feedback. Peptides (2023) 163:170963. doi: 10.1016/j.peptides.2023.170963 36740189 PMC10516609

[34] Retana-Marquez S Juarez-Rojas L Avila-Quintero A Rojas-Maya S Perera G Casillas F. Neuroendocrine disruption is associated to infertility in chronically stressed female rats. Reprod Biol (2020) 20(4):474–83. doi: 10.1016/j.repbio.2020.07.011 32807716

[35] Borrow AP Heck AL Miller AM Sheng JA Stover SA Daniels RM. Chronic variable stress alters hypothalamic-pituitary-adrenal axis function in the female mouse. Physiol Behav (2019) 209:112613. doi: 10.1016/j.physbeh.2019.112613 31299374 PMC6693655

[36] Kreisman MJ McCosh RB Tian K Song CI Breen KM. Estradiol enables chronic corticosterone to inhibit pulsatile luteinizing hormone secretion and suppress kiss1 neuronal activation in female mice. Neuroendocrinology (2020) 110(6):501–16. doi: 10.1159/000502978 PMC7048652 31461711

[37] Nicolaides NC Charmandari E Kino T Chrousos GP. Stress-related and circadian secretion and target tissue actions of glucocorticoids: impact on health. Front Endocrinol (Lausanne) (2017) 8:70. doi: 10.3389/fendo.2017.00070 28503165 PMC5408025

[38] Russell G Lightman S. The human stress response. Nat Rev Endocrinol (2019) 15(9):525–34. doi: 10.1038/s41574-019-0228-0 31249398

[39] Ma X-M Levy A Lightman SL. Emergence of an isolated arginine vasopressin (AVP) response to stress after repeated restraint: a study of both AVP and corticotropin-releasing hormone messenger ribonucleic acid (RNA) and heteronuclear RNA. Endocrinology (1997) 138(10):4351–7. doi: 10.1210/endo.138.10.5446 9322950

[40] Ma X-M Lightman SL Aguilera G. Vasopressin and corticotropin-releasing hormone gene responses to novel stress in rats adapted to repeated restraint. Endocrinology (1999) 140(8):3623–32. doi: 10.1210/endo.140.8.6943 10433220

[41] Figueiredo HF Bodie BL Tauchi M Dolgas CM Herman JP. Stress integration after acute and chronic predator stress: differential activation of central stress circuitry and sensitization of the hypothalamo-pituitary-adrenocortical axis. Endocrinology (2003) 144(12):5249–58. doi: 10.1210/en.2003-0713 12960031

[42] Takumi K Iijima N Higo S Ozawa H. Immunohistochemical analysis of the colocalization of corticotropin-releasing hormone receptor and glucocorticoid receptor in kisspeptin neurons in the hypothalamus of female rats. Neurosci Lett (2012) 531(1):40–5. doi: 10.1016/j.neulet.2012.10.010 23069671

[43] Phumsatitpong C Moenter SM. Estradiol-dependent stimulation and suppression of gonadotropin-releasing hormone neuron firing activity by corticotropin-releasing hormone in female mice. Endocrinology (2018) 159(1):414–25. doi: 10.1210/en.2017-00747 PMC5761586 29069304

[44] Dedic N Kuhne C Jakovcevski M Hartmann J Genewsky AJ Gomes KS. Chronic CRH depletion from GABAergic, long-range projection neurons in the extended amygdala reduces dopamine release and increases anxiety. Nat Neurosci (2018) 21(6):803–7. doi: 10.1038/s41593-018-0151-z PMC6951443 29786085

[45] Ono D Mukai Y Hung CJ Chowdhury S Sugiyama T Yamanaka A. The mammalian circadian pacemaker regulates wakefulness via CRF neurons in the paraventricular nucleus of the hypothalamus. Sci Adv (2020) 6(45):eabd0384. doi: 10.1126/sciadv.abd0384 33158870 PMC7673716

[46] Fuzesi T Daviu N Wamsteeker Cusulin JI Bonin RP Bains JS. Hypothalamic CRH neurons orchestrate complex behaviours after stress. Nat Commun (2016) 7:11937. doi: 10.1038/ncomms11937 27306314 PMC4912635

[47] Herman JP Adams D Prewitt C. Regulatory changes in neuroendocrine stress-integrative circuitry produced by a variable stress paradigm. Neuroendocrinology (1995) 61(2):180–90. doi: 10.1159/000126839 7753337

[48] Prewitt CM Herman JP. Hypothalamo-pituitary-adrenocortical regulation following lesions of the central nucleus of the amygdala. Stress (1997) 1(4):263–80. doi: 10.3109/10253899709013746 9787250

[49] Solomon MB Jones K Packard BA Herman JP. The medial amygdala modulates body weight but not neuroendocrine responses to chronic stress. J Neuroendocrinol (2010) 22(1):13–23. doi: 10.1111/j.1365-2826.2009.01933.x 19912476

[50] Mamalaki E Kvetnansky R Brady LS Gold PW Herkenham M. Repeated immobilization stress alters tyrosine hydroxylase, corticotropin-releasing hormone and corticosteroid receptor messenger ribonucleic Acid levels in rat brain. J Neuroendocrinol (1992) 4(6):689–99. doi: 10.1111/j.1365-2826.1992.tb00220.x 21554656

[51] Shahanoor Z Sultana R Baker MR Romeo RD. Neuroendocrine stress reactivity of male C57BL/6N mice following chronic oral corticosterone exposure during adulthood or adolescence. Psychoneuroendocrinology (2017) 86:218–24. doi: 10.1016/j.psyneuen.2017.10.001 29020649

[52] Stanton LM Price AJ Manning EE. Hypothalamic corticotrophin releasing hormone neurons in stress-induced psychopathology: Revaluation of synaptic contributions. J Neuroendocrinol (2023) 35(4):e13268. doi: 10.1111/jne.13268 37078436

[53] Cayupe B Troncoso B Morgan C Sáez-Briones P Sotomayor-Zárate R Constandil L. The role of the paraventricular-coerulear network on the programming of hypertension by prenatal undernutrition. Int J Mol Sci (2022) 23(19):11965. doi: 10.3390/ijms231911965 36233268 PMC9569920

